# Supplementary material for: Synthesis and superconductivity in yttrium-cerium hydrides at high pressures
Source: Nat Commun. 2024 Feb 28;15:1809. doi: 10.1038/s41467-024-46133-x (PMC10901869; doi:10.1038/s41467-024-46133-x)
Supplement: Supplementary file 1 — Supplementary Information [file 41467_2024_46133_MOESM1_ESM.pdf]

Supplementary Information

Synthesis and superconductivity in yttrium-cerium hydrides at high pressures

Liu-Cheng Chen[1,2], Tao Luo[1,2], Zi-Yu Cao[3,2], Philip Dalladay-Simpson[2], Ge Huang[2], Di Peng[2], Li-Li Zhang[4], Federico Aiace Gorelli[2,5], Guo-Hua Zhong[6,7], Hai-Qing Lin[8], & Xiao-Jia Chen[9⋆]

[1]*School of Science, Harbin Institute of Technology, Shenzhen 518055, China*

[2]*Center for High Pressure Science and Technology Advanced Research, Shanghai 201203, China*

[3]*Center for Quantum Materials and Superconductivity (CQMS) and Department of Physics, Sungkyunkwan University, Suwon 16419, Republic of Korea*

[4]*Shanghai Synchrotron Radiation Facility, Shanghai Advanced Research Institute, Chinese Academy of Sciences, Shanghai 201204, China*

[5]*National Institute of Optics (INO-CNR) and European Laboratory for Non-Linear Spectroscopy (LENS), Via N. Carrara 1, 50019 Sesto Fiorentino (Florence), Italy*

[6]*Shenzhen Institute of Advanced Technology, Chinese Academy of Sciences, Shenzhen 518055, China*

[7]*University of Chinese Academy of Sciences, Beijing 100049, China*

[8]*School of Physics, Zhejiang University, Hangzhou 310058, China*

[9]*Department of Physics and Texas Center for Superconductivity, University of Houston, Houston, TX 77204, USA*

⋆*e-mail: xjchen@uh.edu*

Supplementary Table 1 | Detailed experimental parameters of the DACs in this study (SC: Superconductivity detected from the electrical transport measurements; MF: Electrical transport measurements at external magnetic fields; Raman: Raman scattering measurements; and XRD: X-ray diffraction measurements).

**Number**	**Culet** (μm)	**Gasket**	**Composition**	**Temperature** (K)	**Pressure** (GPa)	**Measurement**
**Cell-1**	100	Re+cBN/epoxy	$Y_{0.5}Ce_{0.5}$+NH_3BH_3	1500-2000	120	SC, Raman, XRD
**Cell-2**	80	Re+cBN/epoxy	$Y_{0.5}Ce_{0.5}$+NH_3BH_3	1500-2000	115	Raman
**Cell-3**	80	Re+cBN/epoxy	$Y_{0.5}Ce_{0.5}$+NH_3BH_3	1500-2000	114	SC, Raman
**Cell-4**	80	Re+cBN/epoxy	$Y_{0.5}Ce_{0.5}$+NH_3BH_3	1500-2000	124	SC, Raman
**Cell-5**	80	Re+cBN/epoxy	$Y_{0.5}Ce_{0.5}$+NH_3BH_3	1500-2000	98-140	SC, Raman, XRD
**Cell-6**	60	Re+cBN/epoxy	$Y_{0.5}Ce_{0.5}$+NH_3BH_3	1500-2000	166-200	SC, Raman
**Cell-7**	80	Re+cBN/epoxy	$Y_{0.5}Ce_{0.5}$+NH_3BH_3	1500-2000	124-155	SC, Raman, MF

Supplementary Table 2 | The obtained lattice parameters (a, c), volume (V), and hydrogen concentration (x) for Cell-1 and Cell-6 at measured pressure. The background was subtracted with the default parameters when being converted the XRD patterns by using Dioptas[1]. The background type of legendre polynomials is employed with the background number of terms of 5. The Le Bail method[2] was used to obtain the structural parameters from the collected XRD data of Cell-1 (Point-1). The Pseudo-Voigt function was selected to fit the peak shape. The obtained lattice parameters are GW = 8.864, GU = -5.202, GV = -7.215, LX = 0.089, and LY = 4.104. The refined factors are R_{wp}=12.1% and R_p = 13.5%. For Cell-1 (Point-2) and Cell-2 (Point-1 and Point-2), we have the acceptable errors in the model fitting.

Cell	Position	Structure	a (Å)	c (Å)	V (Å^3)	x	Pressure (GPa)
1	Point-1	$P6_3/mmc$-$Y_{0.5}Ce_{0.5}H_x$	3.653(5)	5.476(6)	63.33(4)	8.5	128
1	Point-2	$P6_3/mmc$-$Y_{0.5}Ce_{0.5}H_x$	3.642(4)	5.446(6)	62.60(3)	8.3	128
6	Point-1	$P6_3/mmc$-$Y_{0.5}Ce_{0.5}H_x$	3.635(4)	5.373(5)	61.53(3)	8.5	140
6	Point-2	$P6_3/mmc$-$Y_{0.5}Ce_{0.5}H_x$	3.645(6)	5.421(4)	62.41(4)	8.8	140

Supplementary Table 3 | Calculated T_c for $Y_{0.5}Ce_{0.5}H_9$ at 180 GPa with various values of the Coulomb pseudopotential parameter μ^*. The T_c value was calculated from the Allen-Dynes–modified McMillan formula[3] $T_c = \frac{\omega_{log}}{1.2} \exp[-\frac{1.04(1+\lambda)}{\lambda-\mu^*(1+0.62\lambda)}]$ with $\omega_{log} = \exp[\frac{2}{\lambda} \int \ln(\omega) \frac{\alpha^2 F(\omega)}{\omega} d\omega]$ and $\lambda = 2\int \frac{\alpha^2 F(\omega)}{\omega} d\omega$, where $\alpha^2 F(\omega)$ and λ are the electron-phonon spectral function and electron-phonon coupling parameter, respectively.

μ^*	0.10	0.11	0.12	0.13	0.14	0.15
T_c (K)	119.3	116.9	114.5	112.0	106.5	104.2

Supplementary Table 4 | Comparisons of the synthetic pressure, laser heating temperature, stable pressure and T_c of the current work with YH_9[4,5] and CeH_9[6,7] from the literature.

Phases	Synthetic pressure (GPa)	Heating temperature (K)	Stable pressure (GPa)	T_c (K)	Reference
$P6_3/mmc$-YH_9	134-187	Without heating (Catalytic)	134-187	∼216-256	[4]
$P6_3/mmc$-YH_9	Starting from 184	∼1500-2000	185-255	∼230-243	[5]
$P6_3/mmc$-CeH_9	∼90	∼1500	88-155	∼57-100	[6,7]
$P6_3/mmc$-$Y_{0.5}Ce_{0.5}H_9$	∼98-124	1500-2000	98-166	∼97-141	This work

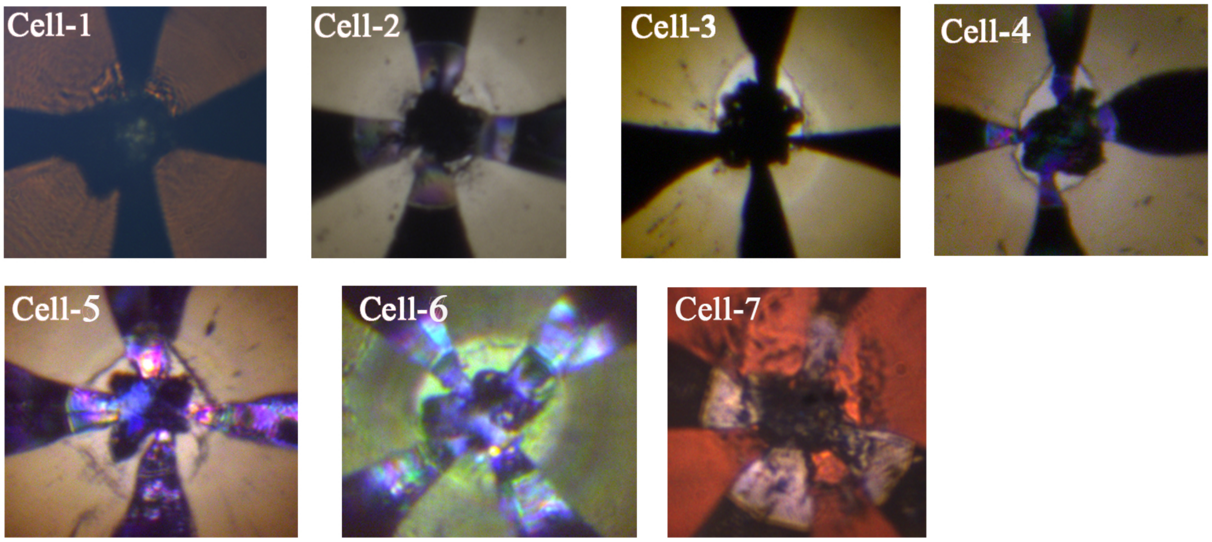

Supplementary Figure 1 | Microphotographs of the samples after laser heating in the chamber of Cell-1 to Cell-7. The sample chamber contains $Y_{0.5}Ce_{0.5}$ connecting well with four Pt leads and the hydrogen source NH_3BH_3 (pressure transmitting medium).

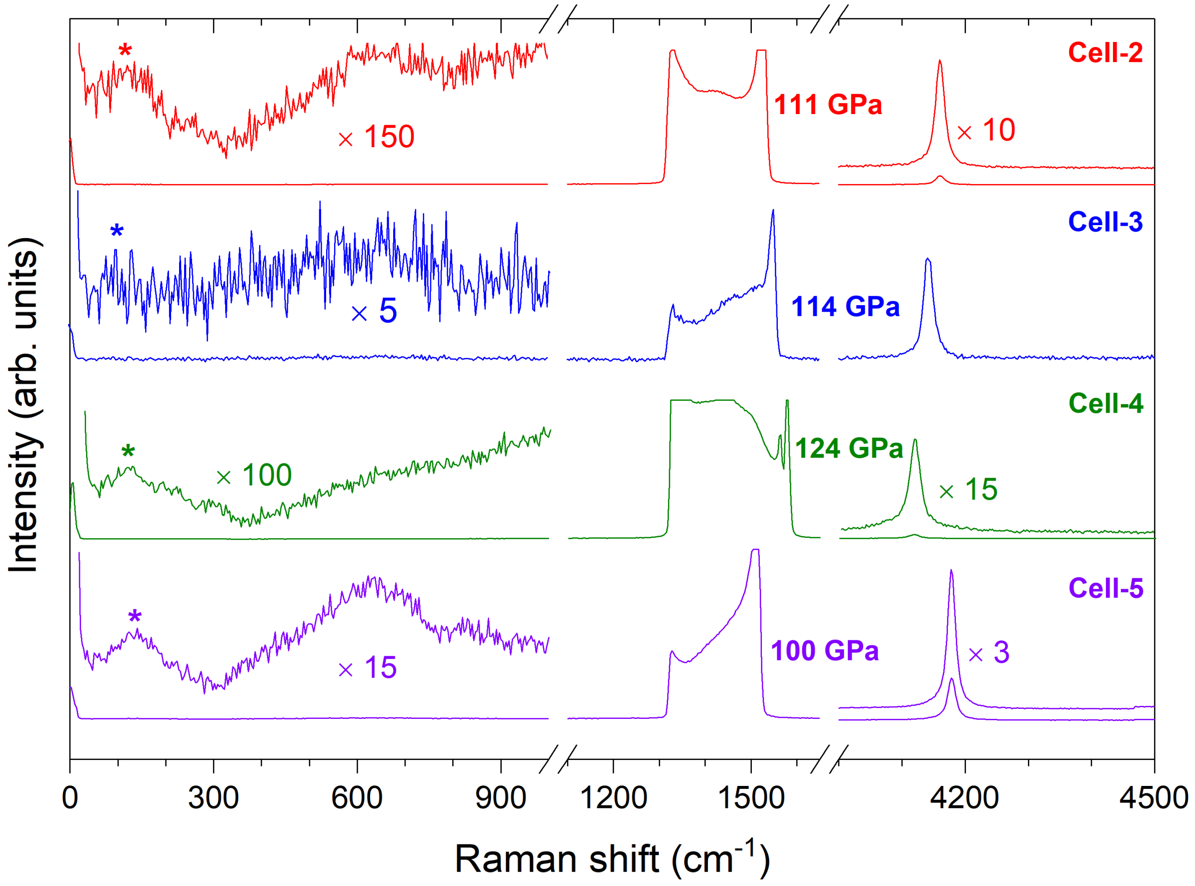

Supplementary Figure 2 | Raman spectra of $Y_{0.5}Ce_{0.5}$ superhydrides after laser heating in Cell-2, Cell-3, Cell-4, and Cell-5 at various pressures. The Raman spectra display the bands from the sample, diamond, and H_2 after laser heating. The low-frequency Raman spectra are scaled for the clarity. The Raman peaks marked with asterisks are the fingerprint for the synthesized Y-Ce hydrides.

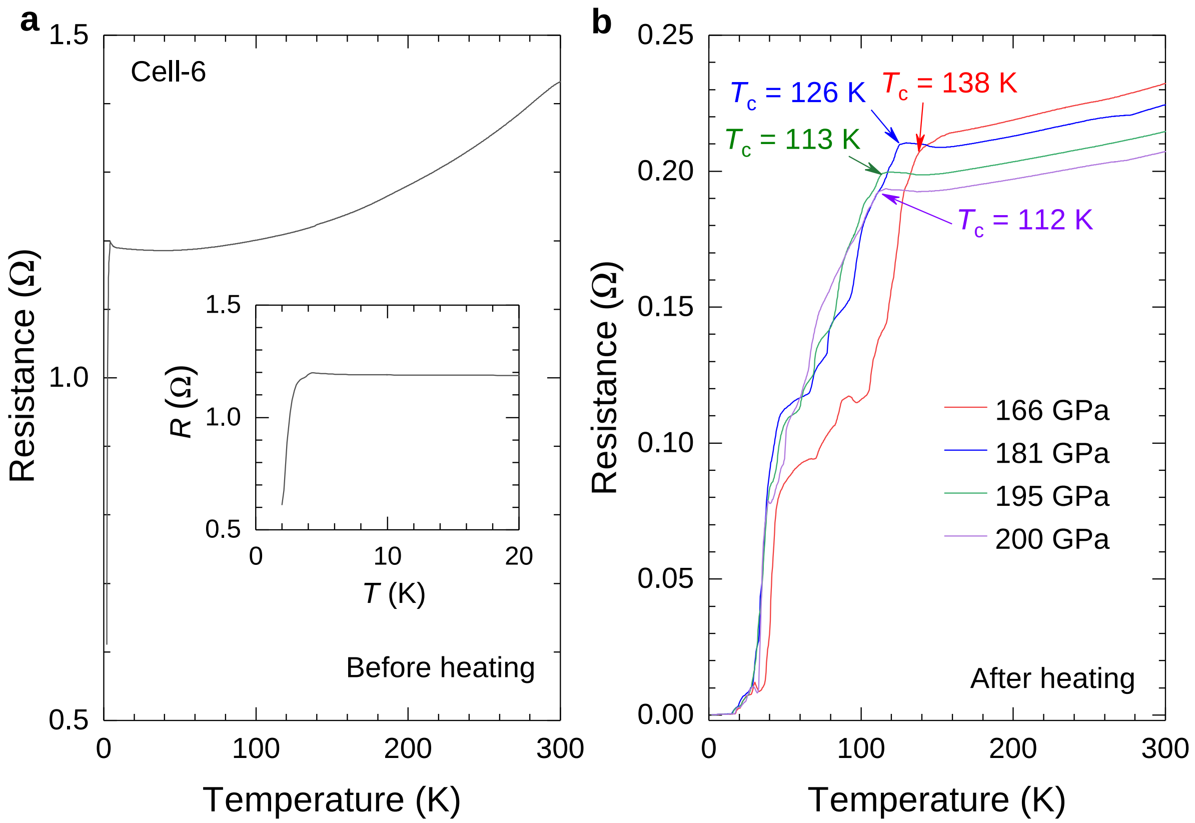

Supplementary Figure 3 | Electrical resistance data of sample 6 (Cell-6) at various pressures. **a**, Temperature-dependent resistance of $Y_{0.5}Ce_{0.5}$ alloy at the pressure of 128 GPa before heating. Inset: Enlarged resistance data in the temperature range of 0-20 K. **b**, Temperature dependence of the resistance of $Y_{0.5}Ce_{0.5}$ hydrides at high pressures after laser heating.

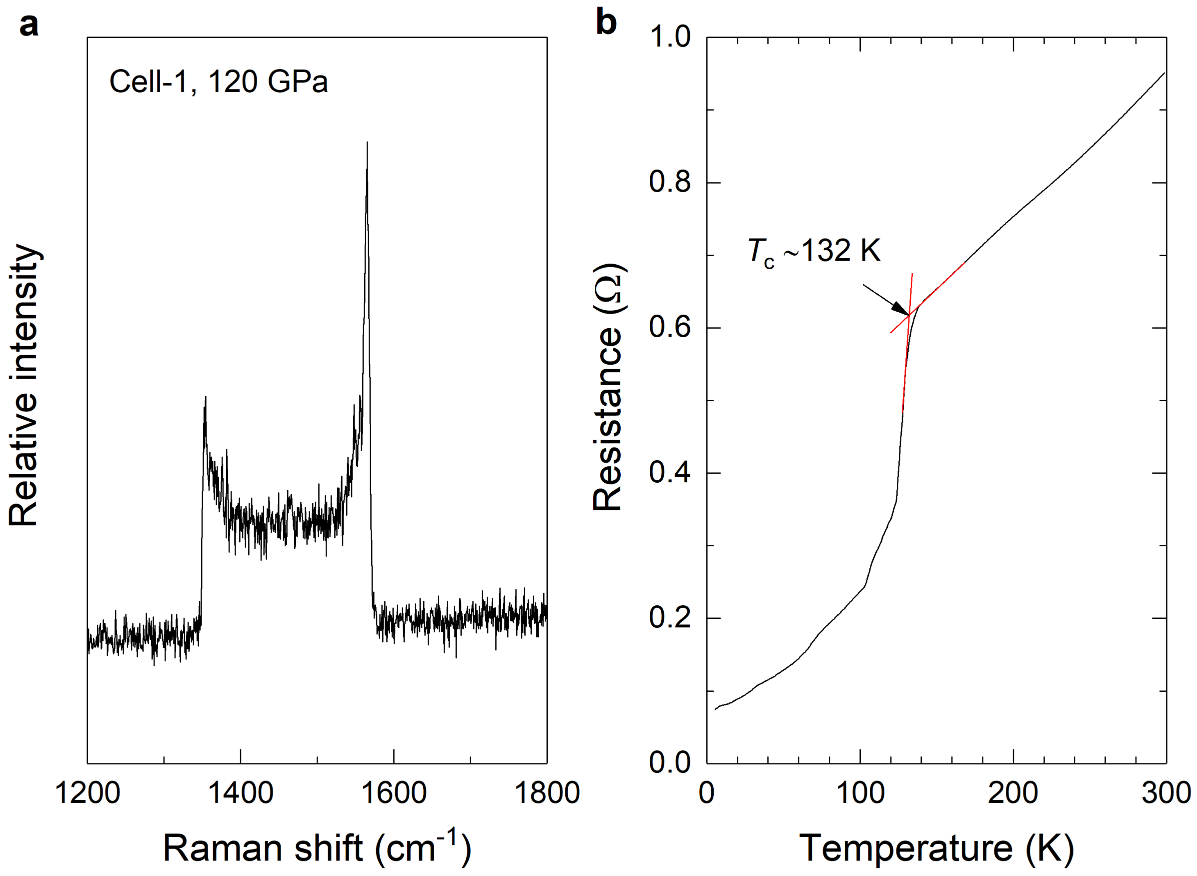

Supplementary Figure 4 | Electrical resistance data of Cell-1 at determined pressure level. a, Raman spectra of diamond used for the pressure calibration. **b**, Resistance data of the sample at 120 GPa after laser heating. The crossing lines show the criterion for determining the superconducting transition temperature T_c.

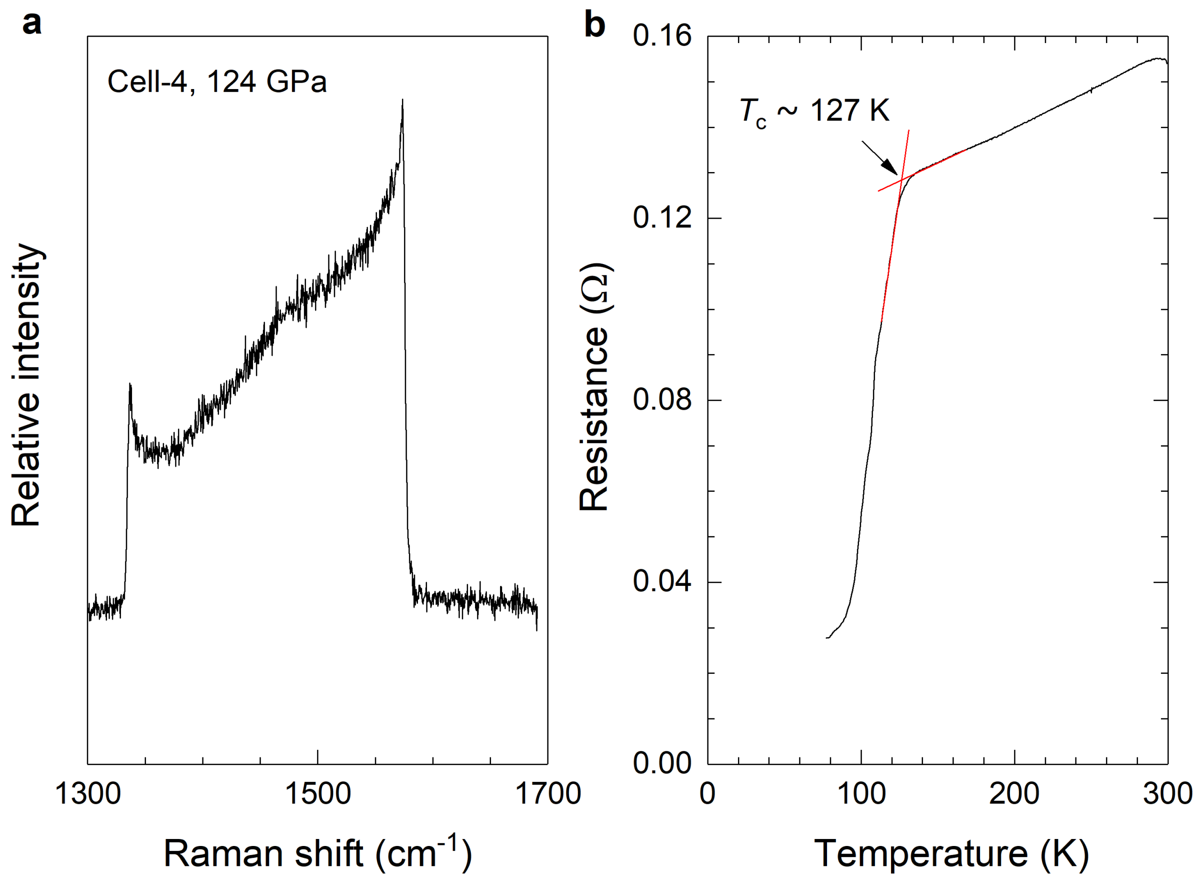

Supplementary Figure 5 | Electrical resistance data of Cell-4 at determined pressure level. a, Raman spectra of diamond used for the pressure calibration. **b**, Resistance data of the sample at 124 GPa after laser heating. The crossing lines determine the T_c value of 127 K.

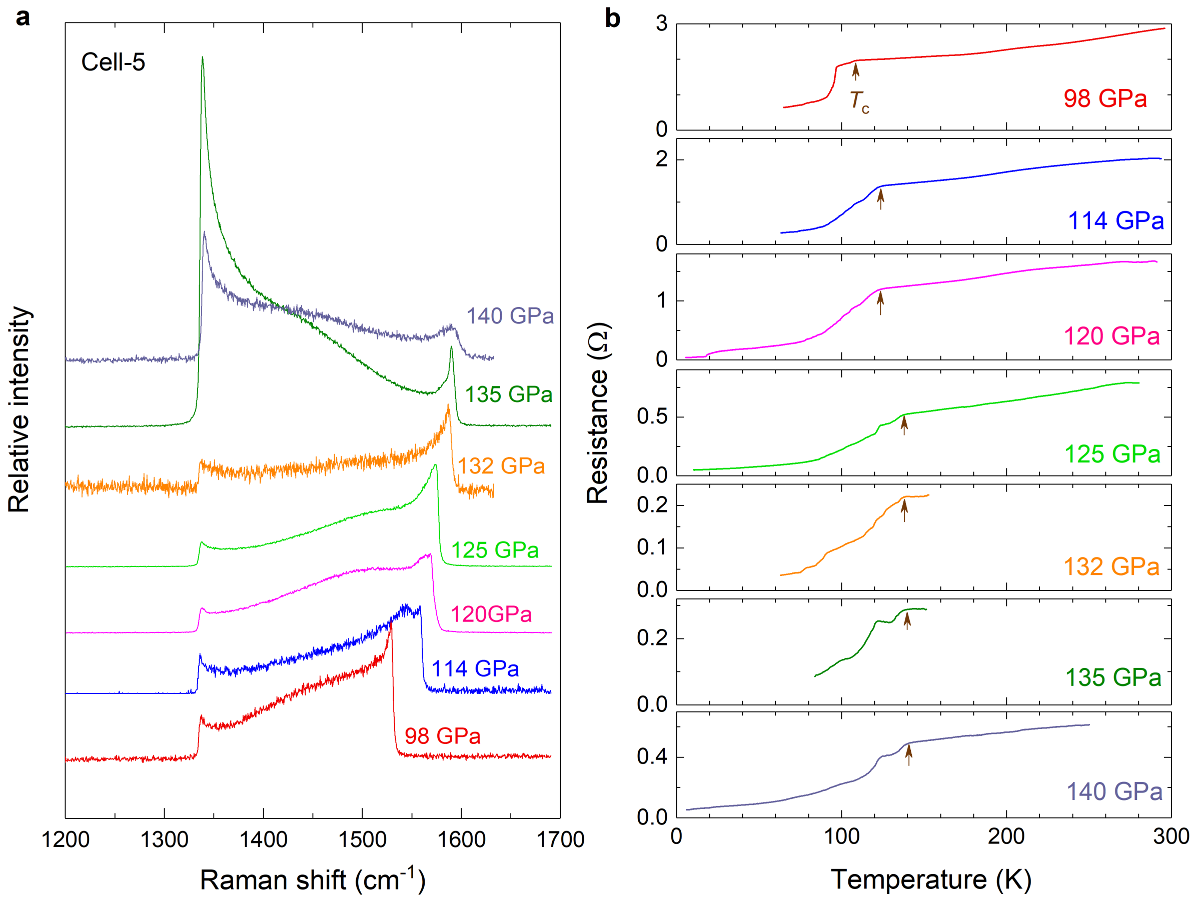

Supplementary Figure 6 | Electrical resistance data of Cell-5 at various pressures. **a**, Raman spectra of diamond used for pressure calibration. **b**, Characterization of the superconducting transition by the electrical resistance measurements under pressure. The arrow denotes the superconducting transition. Some step observed below the superconducting transition for pressure of 125, 135, and 140 GPa is probably due to the pressure gradient and/or geometrical change of the attached leads after laser heating.

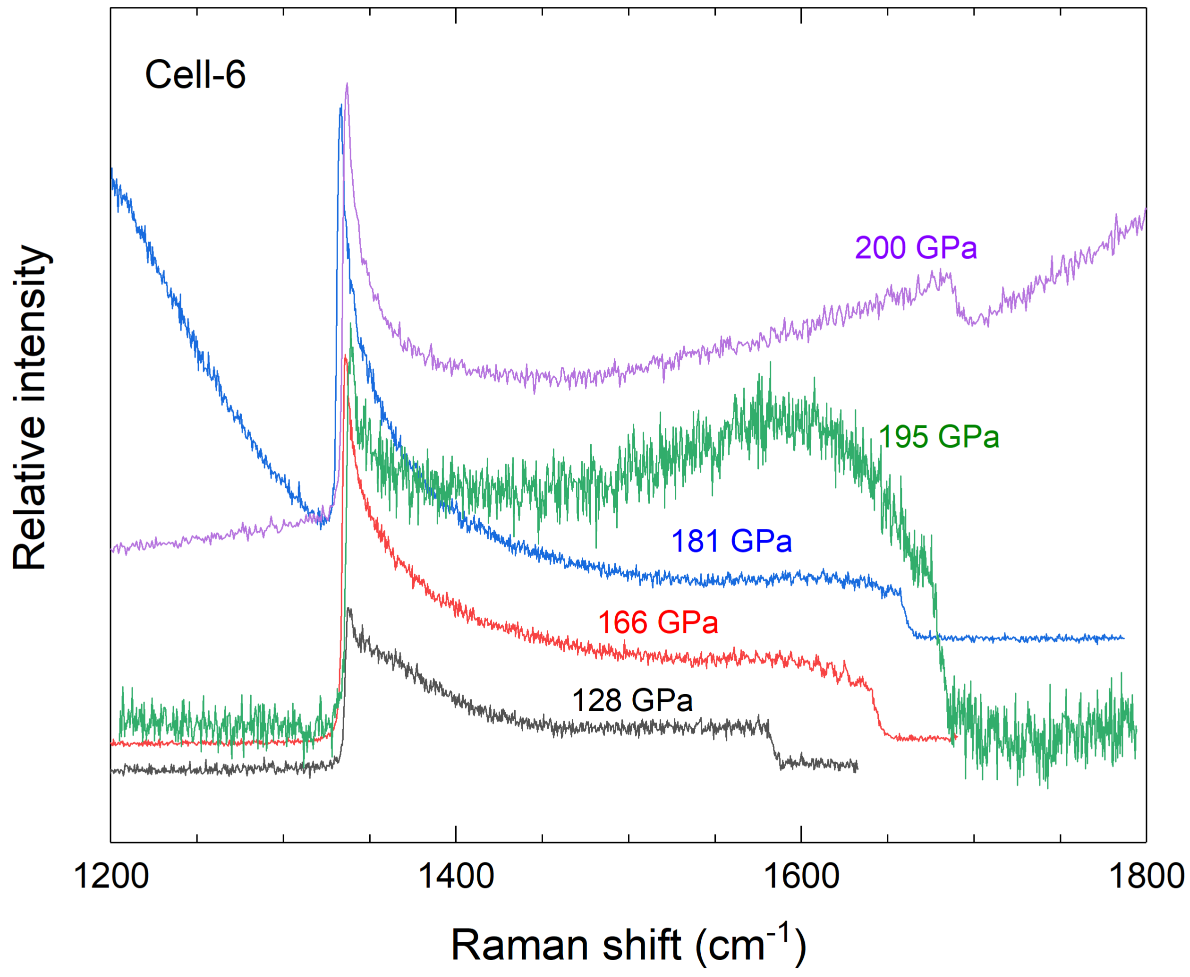

Supplementary Figure 7 | Raman spectra of diamond used for the pressure calibrations in the resistance measurements of sample 6 (Cell-6).

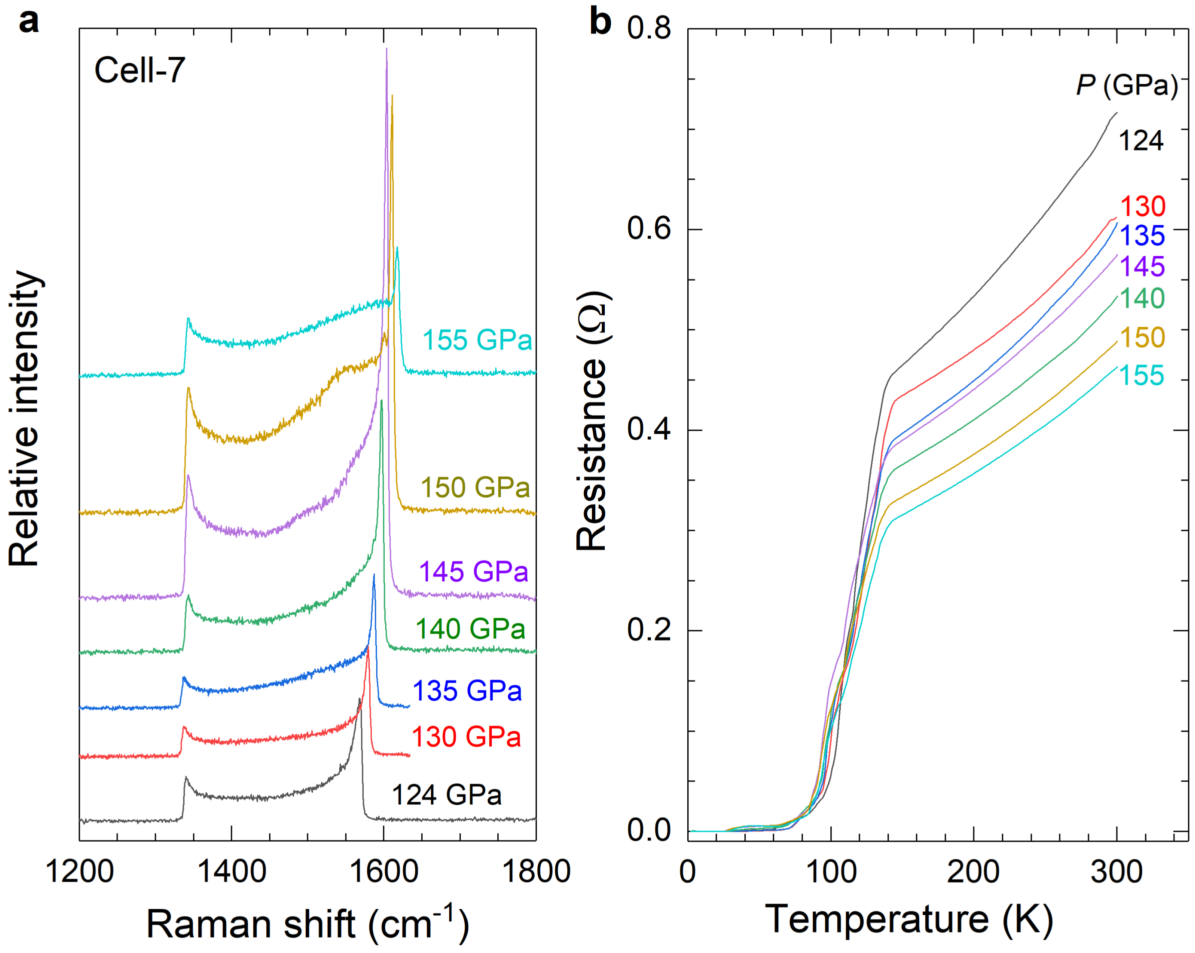

Supplementary Figure 8 | Electrical resistance data of Cell-7 at different pressure levels of sample 7. a, Raman spectra of diamond used for the pressure calibrations. **b**, Temperature dependent resistance of sample 7 at various pressures up to 155 GPa after laser heating.

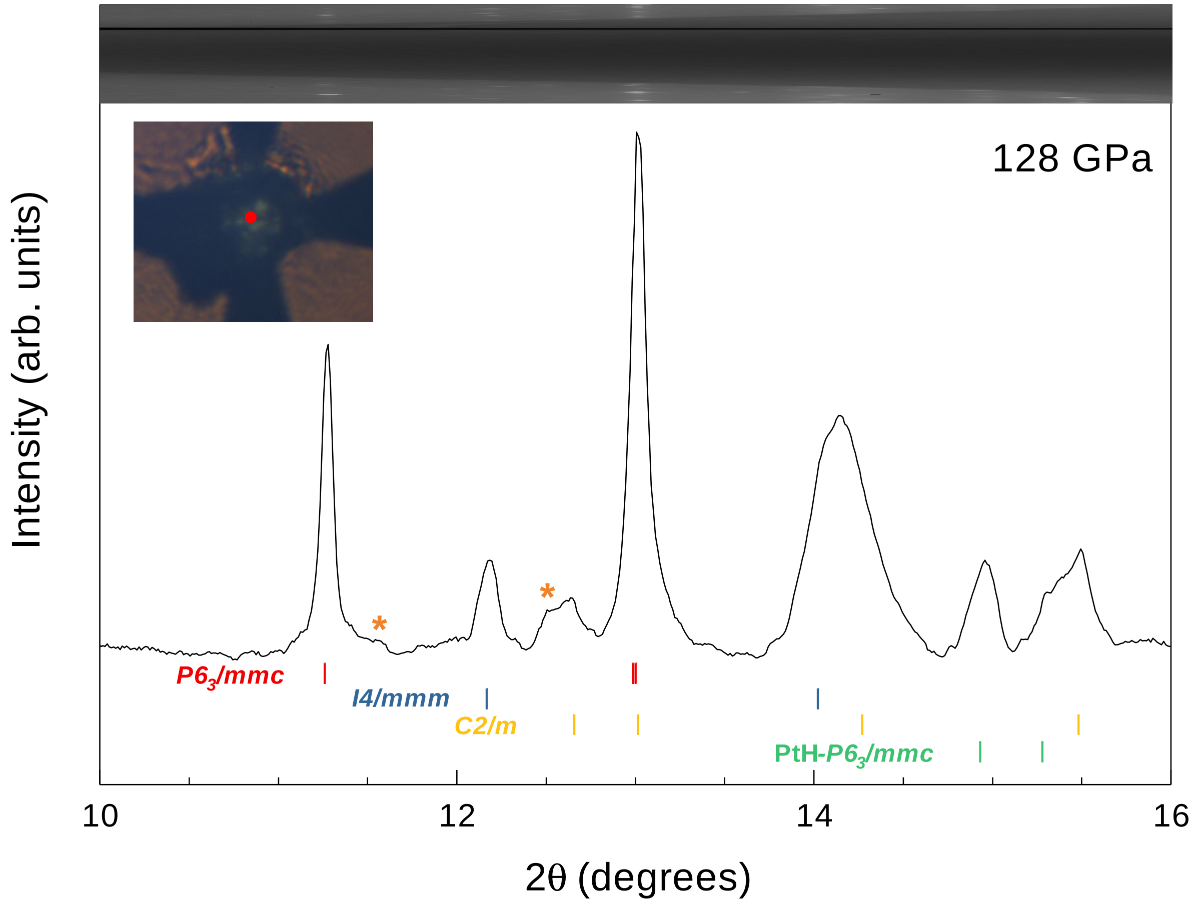

Supplementary Figure 9 | X-ray diffraction patterns of sample 1 at pressure of 128 GPa and room temperature (Point-2). Top panel shows the two-dimensional XRD patterns. The red point in the inset photograph denotes the corresponding sample position of the collected patterns. The sticks at the bottom represent the peaks of the $P6_3/mmc$, $I4/mmm$, and $C2/m$ phase of $Y_{0.5}Ce_{0.5}$ hydrides, and the $P6_3/mmc$ phase of Pt hydride[8]. Unidentified reflections are marked by asterisks.

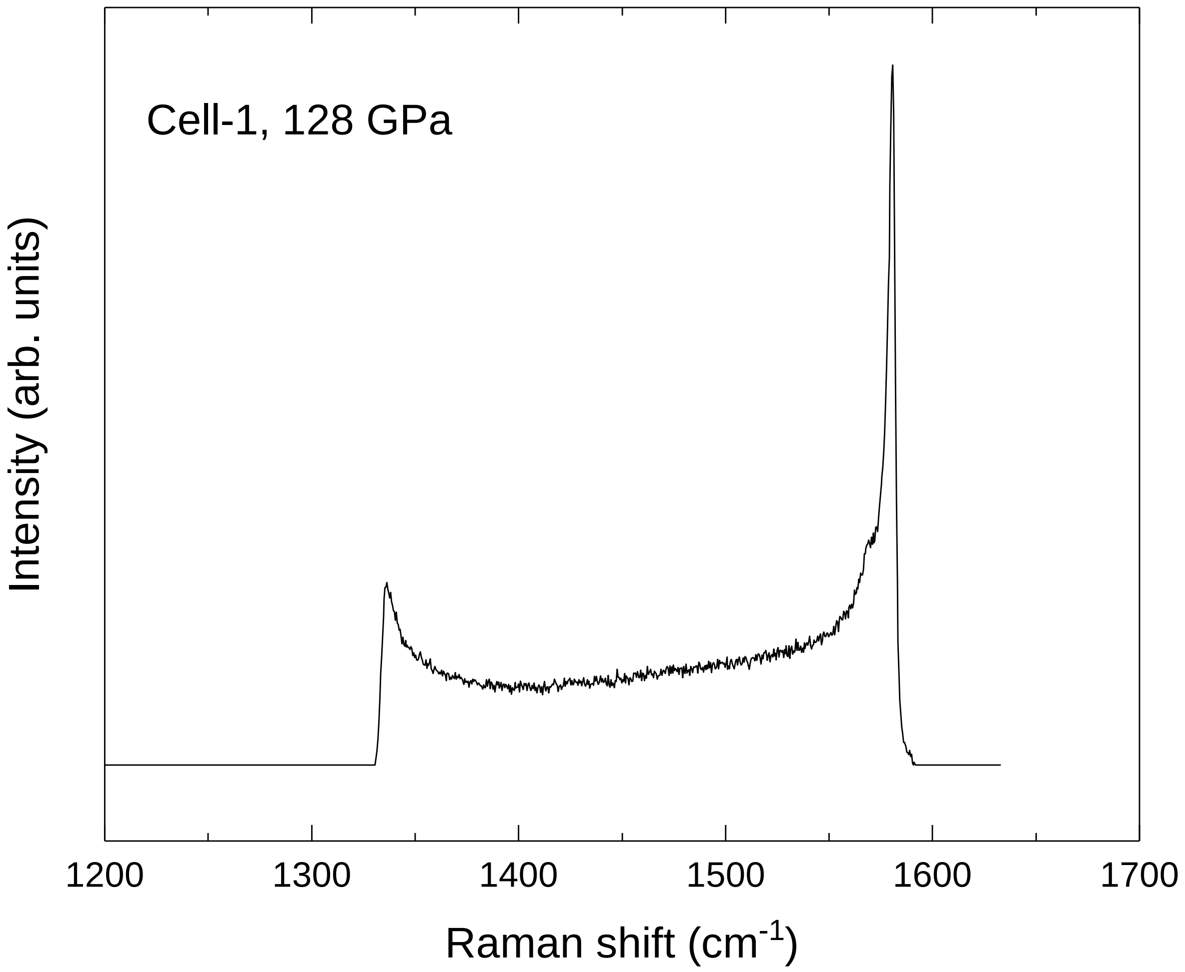

Supplementary Figure 10 | Raman spectra of diamond used for the pressure calibration in the x-ray diffraction measurement of sample 1 (Cell-1).

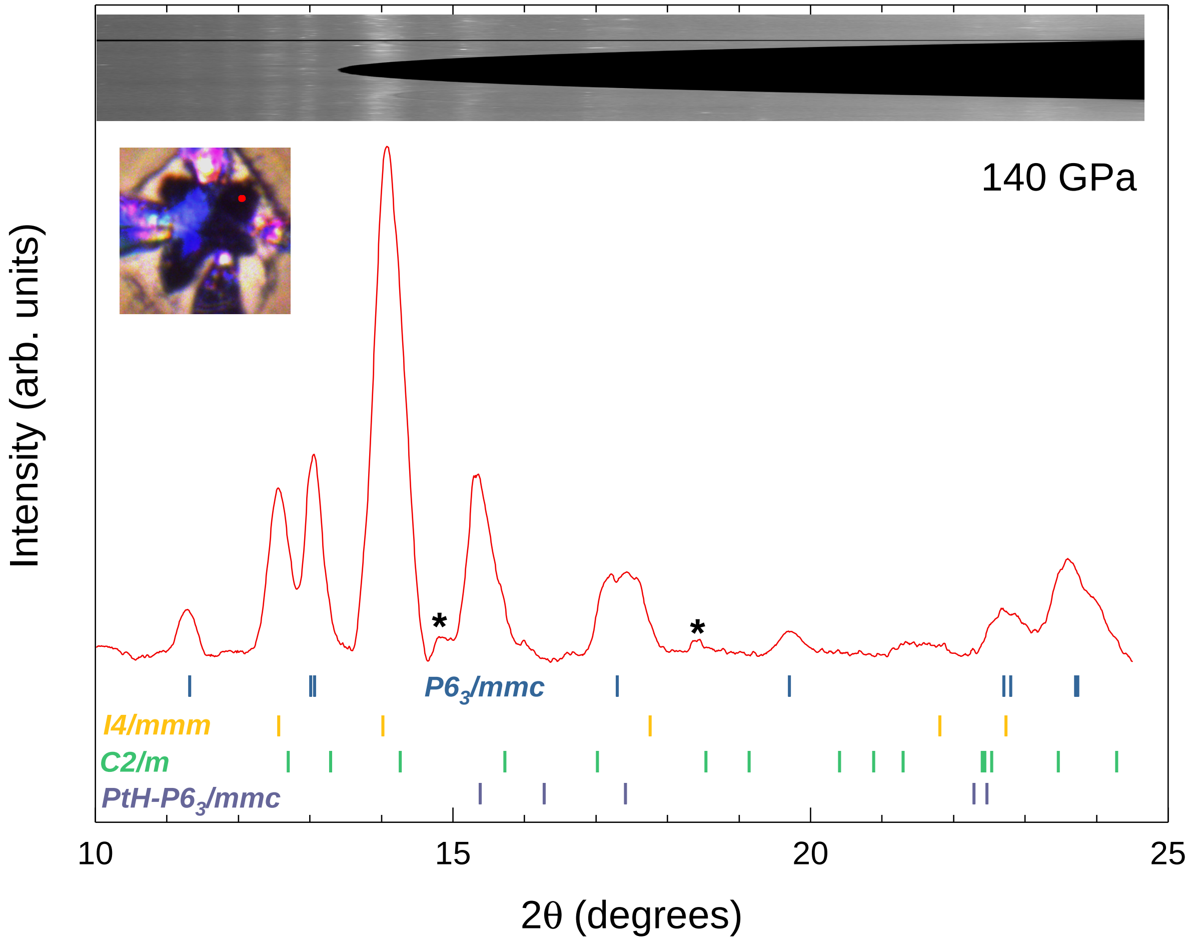

Supplementary Figure 11 | X-ray diffraction patterns of sample 5 (Cell-5) at pressure of 140 GPa (Point-1). Top panel shows the two-dimensional XRD patterns. The red point in the inset photograph refers to the corresponding sample position of the collected patterns. The sticks at the bottom represent the peaks of the $P6_3/mmc$, $I4/mmm$, and $C2/m$ phase of $Y_{0.5}Ce_{0.5}$ hydrides, and the $P6_3/mmc$ phase of Pt hydride[8]. Unidentified reflections are marked by asterisks.

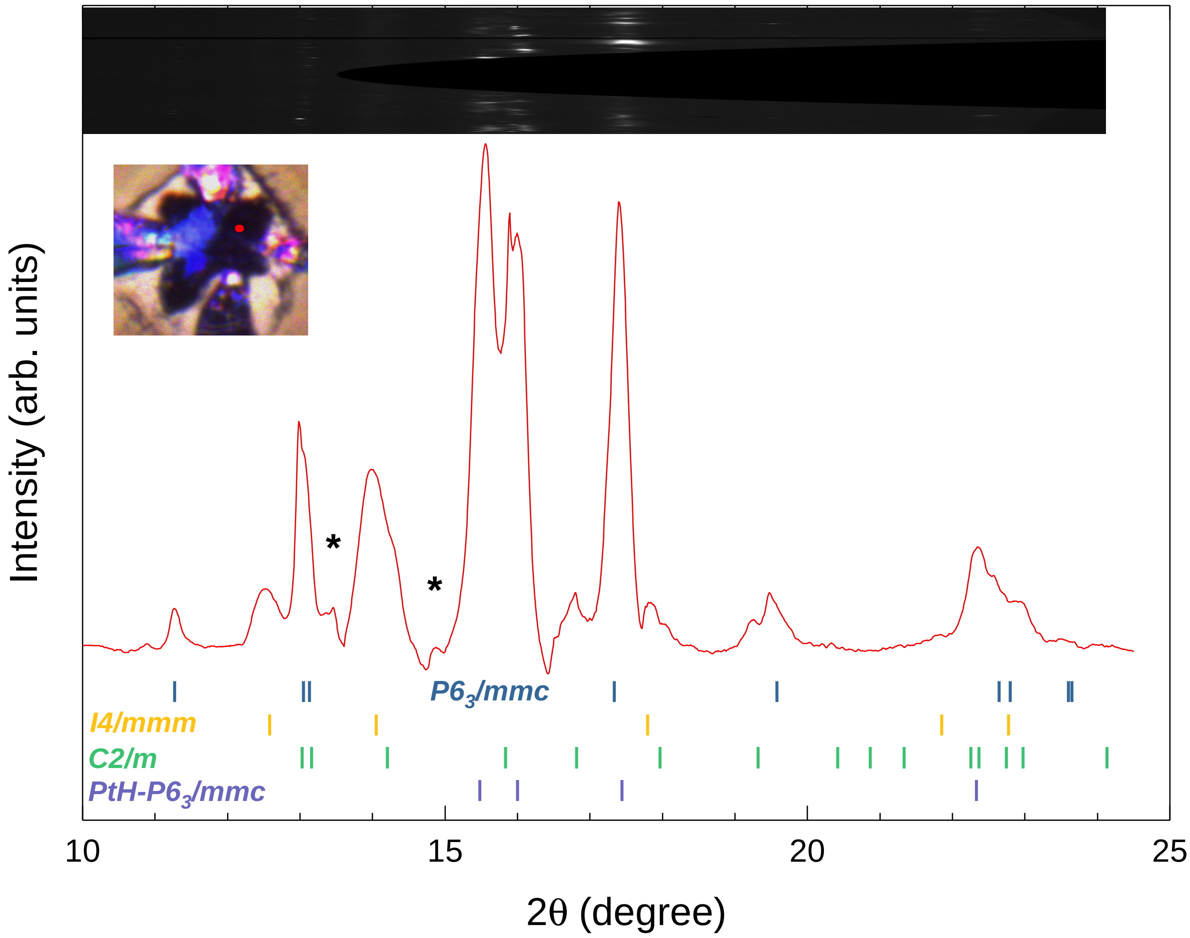

Supplementary Figure 12 | X-ray diffraction patterns of sample 5 (Cell-5) at pressure of 140 GPa (Point-2). Top panel shows the two-dimensional XRD patterns. The red point in the inset photograph denotes the corresponding sample position of the collected patterns. The sticks at the bottom represent the peaks of the $P6_3/mmc$, $I4/mmm$, and $C2/m$ phase of $Y_{0.5}Ce_{0.5}$ hydrides, and the $P6_3/mmc$ phase of Pt hydride[8]. Unidentified reflections are marked by asterisks.

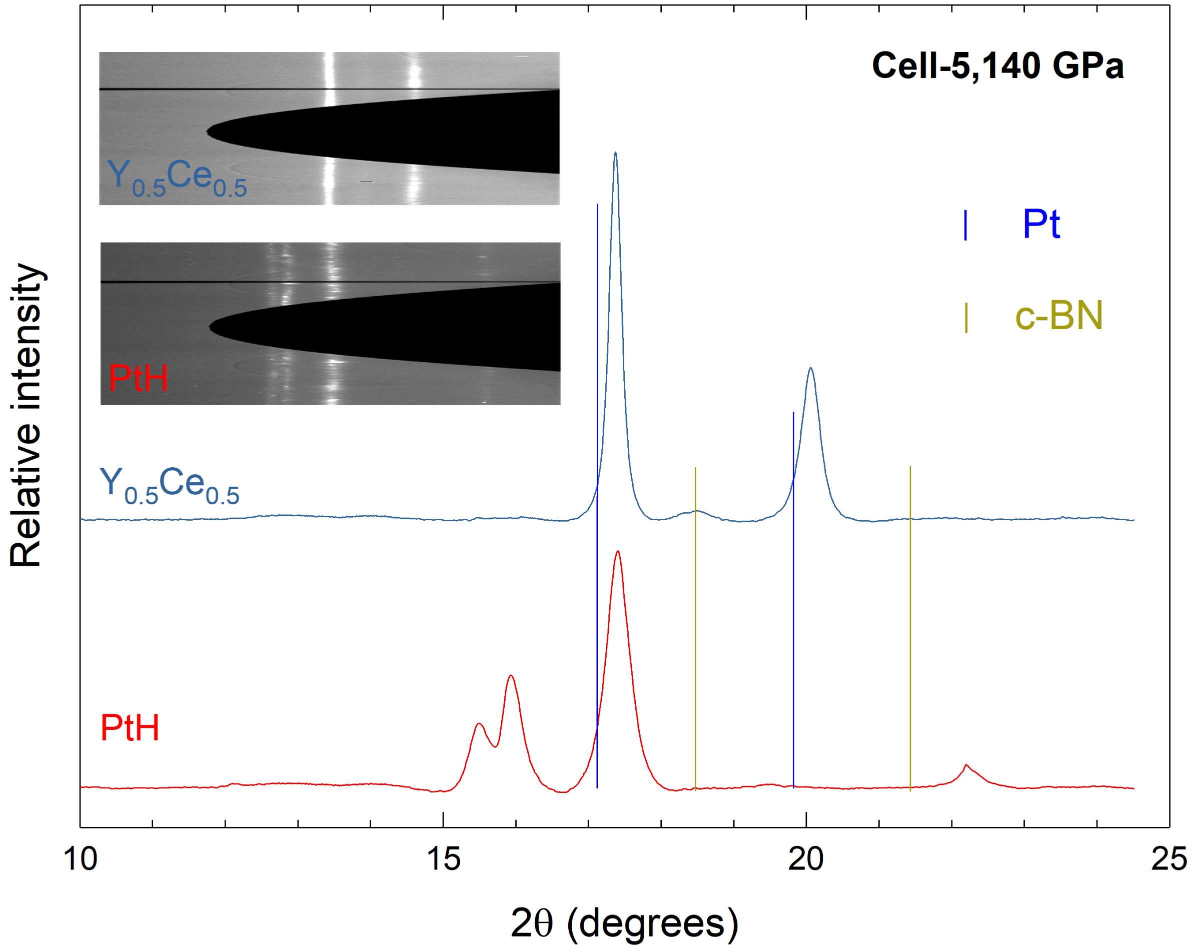

Supplementary Figure 13 | X-ray diffraction patterns of $Y_{0.5}Ce_{0.5}$ alloy and Pt hydride in Cell-5 at pressure of 140 GPa. Insets show the two-dimensional XRD patterns of $Y_{0.5}Ce_{0.5}$ alloy and Pt hydride[8]. The sticks at the bottom represent the peak positions of the phases for Pt and c-BN at around 140 GPa.

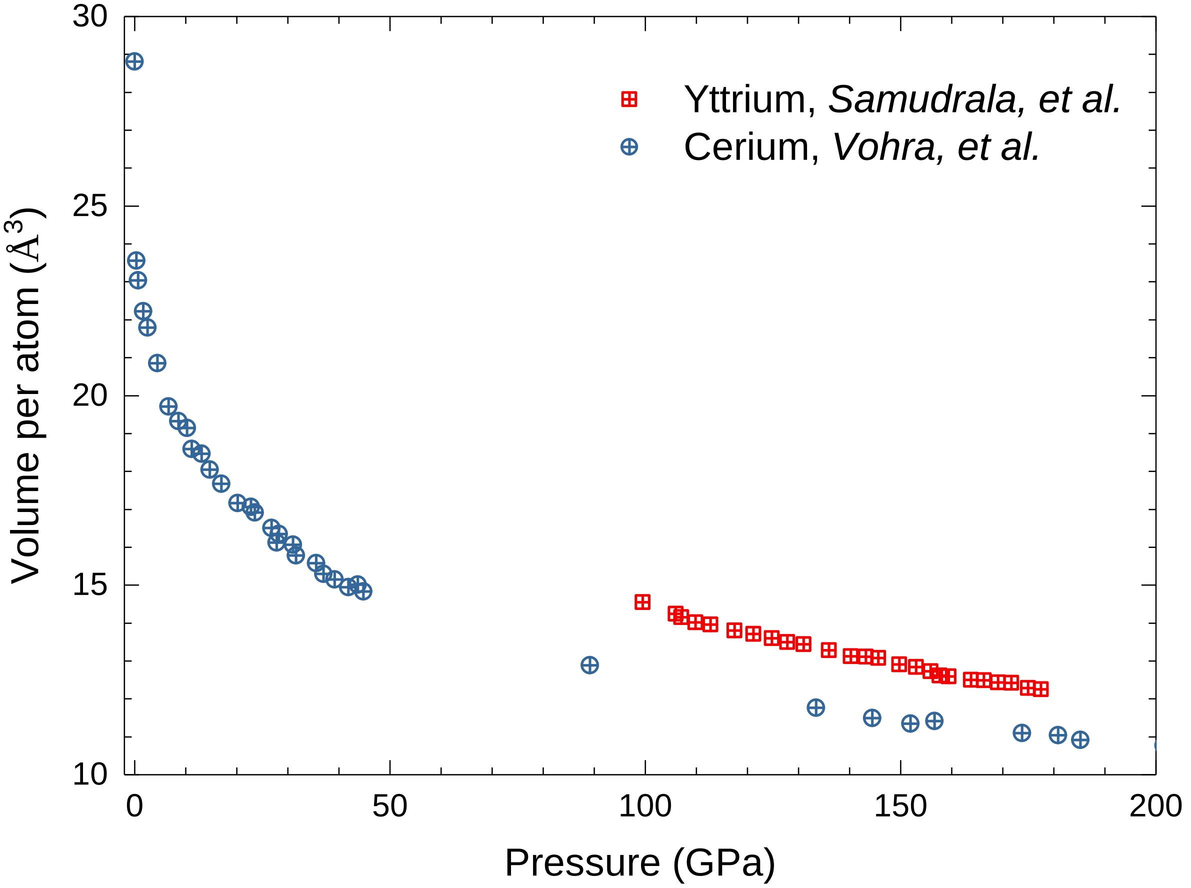

Supplementary Figure 14 | Volume per atom as a function of pressure for yttrium from the work of Samudrala $et\ al.$**[9] and cerium from the work of Vohra** $et\ al.$**[10]**

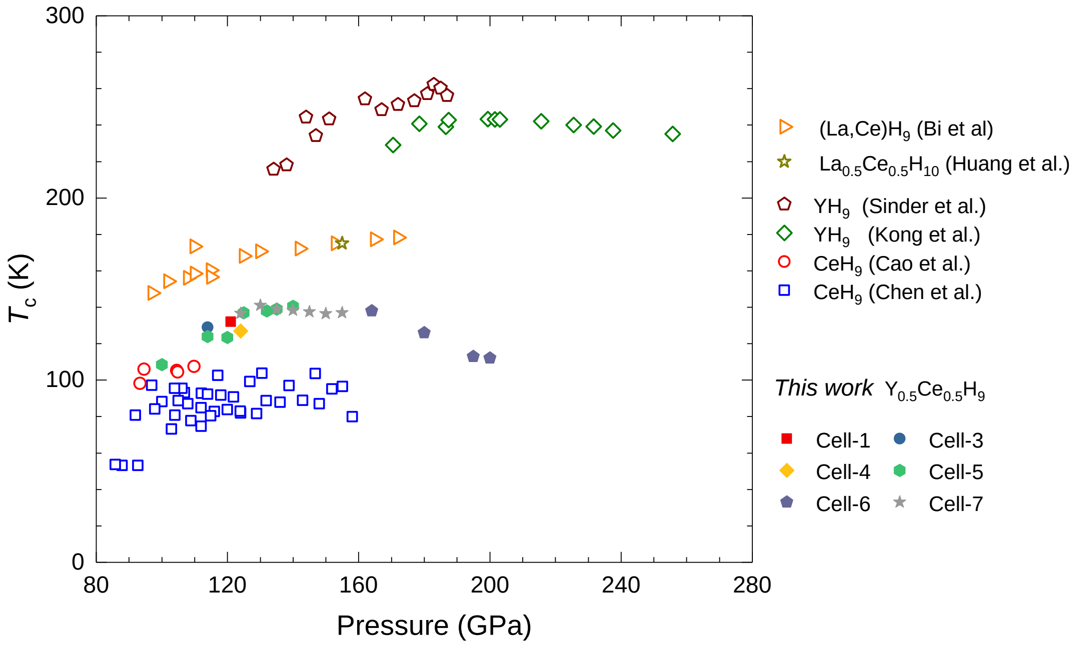

Supplementary Figure 15 | Pressure dependence of T_c of our synthesized $Y_{0.5}Ce_{0.5}H_9$ with the comparison of two ending compounds YH_9 and CeH_9 with the same crystal structure along with the similar systems (La,Ce)H_9 and $La_{0.5}Ce_{0.5}H_{10}$. The solid symbols are our data points in the current work for $Y_{0.5}Ce_{0.5}H_9$ in different runs. The data points for YH_9 are taken from the works of Snider *et al.*[4] and Kong *et al.*[5], respectively. The results for CeH_9 are taken from the works of Chen *et al.*[7] and Cao *et al.*[11]. The experimental data points for (La,Ce)H_9 and $La_{0.5}Ce_{0.5}H_{10}$ are taken from the works of Bi *et al.*[12] and Huang *et al.*[13], respectively.

Supplementary references

1. Prescher, C. & Prakapenka, V. B. Dioptas: A program for reduction of two-dimensional x-ray diffraction data and data exploration, *High Pressure Res.* **35**, 223-230 (2015).

2. Le Bail, A. Whole powder pattern decomposition methods and applications: A retrospection. *Powder diffr.* **20**, 316-326 (2005).

3. Allen, P. B. & Dynes, R. C. Transition temperature of strong-coupled superconductors reanalyzed. *Phys. Rev. B* **12**, 905-922 (1975).

4. Snider, E., Dasenbrock-Gammon, N., McBride, R., Wang, X., Meyers, N., Lawler, K. V., Zurek, E., Salamat, A. & Dias, R. P. Synthesis of yttrium superhydride superconductor with a transition temperature up to 262 K by catalytic hydrogenation at high pressures. *Phys. Rev. Lett.* **126**, 117003 (2021).

5. Kong, P. P. $et\ al$. Superconductivity up to 243 K in the yttrium-hydrogen system under high pressure. *Nat. Commun.* **12**, 5075 (2021).

6. Li, X., Huang, X. L., Duan, D. F., Pickard, C. J., Zhou, D., Xie, H., Zhuang, Q., Huang, Y. P., Zhou, Q., Liu, B. B. & Cui, T. Polyhydride CeH_9 with an atomic-like hydrogen clathrate structure. *Nat. Commun.* **10**, 3461 (2019).

7. Chen, W. H., Semenok, D. V., Huang, X. L., Shu, H. Y., Li, X., Duan, D. F., Cui, T. & Oganov, A. R. High-temperature superconducting phases in cerium superhydride with a T_c up to 115 K below a pressure of 1 megabar. *Phys. Rev. Lett.* **127**, 117001 (2021).

8. Matsuoka, T., Hishida, M., Kuno, K., Hirao, N., Ohishi, Y., Sasaki, S., Takahama, K. & Shimizu, K. Superconductivity of platinum hydride. *Phys. Rev. B* **99**, 144511 (2019).

9. Samudrala, G. K., Tsoi, G. M. & Vohra, Y. K. Structural phase transitions in yttrium under ultrahigh pressures. *J. Phys.: Condens. Matter* **24**, 36220 (2012).

10. Vohra, Y. K., Beaver, S. L., Akella, J., Ruddle, C. A. & Weir., S. T. Ultrapressure equation of state of cerium metal to 208 GPa. *J. App. Phys.* **85**, 2451-2453 (1999).

11. Cao, Z. Y., Choi, S., Chen, L. C., Dalladay-Simpson, P., Jang, H., Gorelli, F. A., Yan, J. F., Jung, S. G., Huang, G., Yu, L., Lee, Y. J., Kim, J. Y., Park, T. & Chen, X. J. Probing superconducting gap in CeH_9 under pressure. *arXiv*: 2401.12682.

12. Bi, J. K., Nakamoto, Y. K., Zhang, P. Y., Shimizu, K., Zou, B., Liu, H. Y., Zhou, M., Liu, G. T., Wang, H. B. & Ma, Y. M. Giant enhancement of superconducting critical temperature in substitutional alloy $(La,Ce)H_9$. *Nat. Commun.* **13**, 5952 (2022).

13. Huang, G., Peng, D., Luo, T., Chen, L. C., Dalladay-Simpson, P., Cao, Z. Y., Gorelli, F. A., Zhong, G. H., Lin, H. Q. & Chen, X. J. Synthesis of superconducting phase of $La_{0.5}Ce_{0.5}H_{10}$ at high pressures. *J. Phys.: Condens. Matter* **36**, 075702 (2024).
